# Cardiometabolic Burden of Irritable Bowel Syndrome: A Case-Control Analysis of Metabolic Syndrome Clustering

**DOI:** 10.1007/s44197-026-00584-1

**Published:** 2026-05-25

**Authors:** Mohammed. N. Abdelaziz, Omar Abdallah, Anwar M. Bshar, Mahmoud Alqenawy, Hager G. Elkasabi, Nada F. Mansour, Tasneem Mahmoud, Merna M. E. Abdelaziz, Ahmed R. A. Moustafa, Ahmed Almenshawy, Basma Z. Elmorsy

**Affiliations:** 1 Faculty of Medicine, Mansoura University, Mansoura, Egypt; 2 Hepatology and Gastroenterology Unit, Department of Internal Medicine, Faculty of Medicine, Mansoura University, Mansoura, Egypt; 3 Clinical Pathology Department, Mansoura University, Mansoura, Egypt; 4 Endocrinology and Diabetes Unit, Internal Medicine Department, Mansoura University, Mansoura, Egypt

**Keywords:** Irritable bowel syndrome, Metabolic syndrome, Lipid profile, Anthropometric measures

## Abstract

**Background:**

Irritable bowel syndrome **(**IBS) is a common functional gastrointestinal (GI) disorder, which is more frequently associated with some extra-intestinal comorbidities, particularly cardiometabolic comorbidities. Metabolic syndrome (MetS) comprises a constellation of risk factors for cardiovascular diseases, including central obesity, dyslipidemia, hypertension, and insulin resistance. Therefore, the purpose of this study was to assess the prevalence and factors associated with MetS in IBS patients.

**Methods:**

A hospital-based cross-sectional case-control study was conducted, enrolling 140 IBS patients diagnosed according to Rome IV criteria and 140 non-IBS controls from a healthcare center in Dakahlia, Egypt. Metabolic syndrome was defined using International Diabetes Federation criteria. Demographic information was collected together with anthropometric measurements and clinical and biochemical data. Binary logistic regression analysis was conducted with sequential models that adjusted for age, sex, and lifestyle factors (body mass index, smoking status, and residence). Model discrimination was evaluated using the area under the receiver operating characteristic (ROC) curve (AUC).

**Results:**

IBS patients showed a significant increase in metabolic syndrome rates at 49.3% compared to 17.1% in controls, p-value < 0.001. The metabolic components of IBS cases showed significant elevation in the following measures: abdominal obesity occurred in 71.4% of cases compared to 44.3% in controls and fasting blood glucose levels ≥ 100 mg/dL occurred in 42.9% of cases compared to 19.3% in controls and blood pressure levels ≥ 130/85 mmHg occurred in 57.9% of cases compared to 17.9% in controls and triglyceride levels ≥ 150 mg/dL occurred in 25.7% of cases compared to 15.7% in controls. Low-density lipoprotein cholesterol levels (median values) were 115 mg/dL in cases versus 102 mg/dL in controls (*p* = 0.01). When age, sex, BMI, smoking status, and residence were considered in the multivariate logistic regression model, IBS status remained an independent predictor of metabolic syndrome (AOR: 7.818; 95% CI: 3.913–15.620; *p* < 0.001). This model demonstrated excellent discriminatory performance (AUC: 0.852; 95% CI: 0.806–0.898) and adequate calibration (Hosmer-Lemeshow: 0.442). Within the IBS cohort, BMI functioned as the only independent predictor for metabolic syndrome co-occurrence (AOR: 1.18 per unit increase; 95% CI: 1.08–1.30; *p* < 0.001), with the model correctly classifying 75.7% of cases (sensitivity: 73.9%; specificity: 77.5%).

**Conclusion:**

Our findings suggest that IBS may identify a subgroup of patients with elevated cardiometabolic burden, warranting routine cardiometabolic evaluation in clinical practice. However, causal relationships and temporal sequence cannot be inferred from this cross‑sectional case–control design.

## Introduction

Irritable bowel syndrome (IBS) is recognized for changes in bowel habits and recurrent episodes of abdominal pain. This disorder is chronic and functional, affecting 10–20% of adults worldwide [1]. A prevalence of 28.9% was reported in Egyptian adults [2]. Thus, this disorder has a considerable impact on health systems, work productivity, and quality of life [1–3]. IBS is increasingly recognized as a systemic disorder with extra-intestinal manifestations that extend beyond gastrointestinal symptoms [4, 5]. Notably, obesity and visceral adiposity are prevalent among IBS patients, suggesting a clinically relevant interface between functional gastrointestinal disorders and cardiometabolic risk [6].

Alongside this, metabolic syndrome (MetS), defined as a combination of conditions including central obesity, dyslipidemia, hypertension, and impaired glucose homeostasis, is increasingly viewed as a major public health challenge given its strong association with type 2 diabetes, cardiovascular morbidity, and all-cause mortality [7, 8]. Notably, obesity and visceral adiposity are key determinants of metabolic syndrome. Given the growing evidence suggesting metabolic alterations among patients with IBS, this overlap may indicate a clinically relevant interface between functional gastrointestinal disorders and cardiometabolic risk [9].

Increasingly, studies are supporting a bidirectional relationship between IBS and MetS, likely due to shared pathophysiological mechanisms, including low-grade systemic inflammation, autonomic dysregulation, and alterations in the gut-brain-microbiota axis [10–12]. Notwithstanding these observations, there are still substantial gaps in knowledge. Most studies have so far concentrated on Asian or Western populations [10], leaving very few data from the Middle East and North Africa, more specifically in Egypt, where the epidemic of obesity and diabetes is rapidly rising [13, 14].

Few studies have sought to identify which independent demographic, clinical, or anthropometric characteristics predict MetS in IBS populations in this setting, and it has not yet been established whether cardiometabolic risk profiles vary significantly across specific IBS subtypes. All these points are necessary to categorize high-risk patients who could benefit from focused screening and early action. The present study was conducted among Egyptian adults visiting a tertiary care center for: The present study was conducted among Egyptian adults visiting a tertiary care center for: (1) assessing the association between IBS and MetS; (2) estimating MetS prevalence and characterizing clinical/laboratory profiles among IBS cases vs. non-IBS controls; and (3) identifying independent predictors of MetS within the IBS cohort.

## Methods and Materials

### Study Design and Settings

From December 2023 to May 2024, this cross-sectional case–control study was conducted in two gastrointestinal outpatient clinics at Mansoura Specialized Medical Hospital. Mansoura Specialized Medical Hospital functions as the primary tertiary referral center for Al-Daqahlia Governorate (~ 6 million population) and surrounding Delta regions, receiving both community referrals and primary care overflow for gastroenterology and internal medicine. This setting ensures Rome IV-confirmed IBS diagnoses while providing non-IBS controls from comparable internal medicine clinics, balancing diagnostic specificity with clinical representativeness. Patients who visited the clinics during the study period were evaluated for enrollment using the Rome IV criteria using a consecutive non-probability sampling procedure. During the same study period, non-IBS control subjects were consecutively recruited from the same center’s general internal medicine outpatient clinics.

### Selection Criteria

The study group included individuals with IBS according to the Rome IV criteria, with various BMIs and weights, and aged 18–50 years. Patients with the following diagnoses were not eligible to participate in the study: (A) refusal to participate; (B) red flag symptoms (unexplained anemia, bloody stool, persistent fever, nocturnal symptoms, steatorrhea, onset of age > 50 years (to exclude colorectal malignancy risk per clinical guidelines), rectal bleeding, significant weight loss, intractable vomiting); (C) age < 18 years; (D) organic GI diseases including inflammatory bowel disease (Crohn’s disease, ulcerative colitis), celiac disease, tumors, cholelithiasis, peptic ulcer disease, GERD, cirrhosis, or other liver diseases; (E) prior GI surgery; and (F) any diagnosed psychiatric disorders (to enhance Rome IV diagnostic specificity by excluding somatic symptom mimics). (G) Patients receiving insulin therapy were excluded to minimize confounding by advanced diabetes and treatment-related metabolic effects, thereby allowing a clearer evaluation of early cardiometabolic alterations associated with IBS.

Inclusion criteria for controls included adults aged 18–50 years without gastrointestinal symptoms meeting Rome IV criteria for IBS or any other functional bowel disorder. Exclusion criteria mirrored those for the IBS group and also included any history of gastrointestinal symptoms (such as abdominal pain or altered bowel habits), organic gastrointestinal disease (e.g., inflammatory bowel disease, celiac disease, colorectal cancer), or prior consultation with a gastroenterologist. Absence of IBS was confirmed through a structured symptom questionnaire administered by trained research staff. All clinical, anthropometric, and laboratory assessments in the control participants followed identical standardized protocols as those in the IBS group to minimize measurement bias.

### Sample Size

The G*Power program (version 3.1.9.7) was used to determine the sample size. In a prior Turkish study by Bayrak [5], the prevalence of metabolic syndrome was 21.7% in the control group and 36.8% in the IBS group. A difference in group proportions of 0.1510 may be detected with 80% power using group sample sizes of 140 in the IBS group and 140 in the control group. According to the alternative hypothesis, the proportion in the IBS group is 0.3680, whereas under the null hypothesis, it is considered to be 0.2170. The control group’s share is 0.2170. The two-sided Z-test with unpooled variance is the statistical test that was employed. The test has a significance level of 0.05. Observed rates exceeded hypothesized prevalence, reflecting regional epidemiologic differences, a tertiary care setting, and Rome IV diagnostic rigor. A post-hoc power yielded an achieved power of 0.99 for detecting the difference between proportions, confirming that the study was more than adequately powered for its primary comparison.

### Clinical and Anthropometric Measurements

Through a structured interviewer-administered questionnaire, medical history evaluation was performed on all individuals, covering sociodemographic data, general patient characteristics, symptoms unique to IBS, and the detection of any other coexisting medical conditions. Regarding dietary patterns, participants were specifically queried during the clinical interview regarding adherence to any predefined dietary regimens, including low-FODMAP, Mediterranean, high-fiber, or gluten-free diets. For each dietary pattern, participants were asked to report current or recent adherence (within the preceding 3–6 months). Adherence was defined as self-reported regular commitment to the specified dietary pattern for a sustained period, rather than occasional or intermittent dietary modifications. Family history of IBS was assessed by participant self-report during structured interviews, defined as having at least one first-degree relative (parent, sibling, or child) with physician-diagnosed IBS. No medical record verification was performed due to logistical constraints in this outpatient setting. Furthermore, all participants underwent abdominal examination. Using properly calibrated equipment, height and weight measurements were taken with the participant attired lightly and without shoes. Body mass index (BMI) was calculated as viable weight (kg) divided by height (m²). Waist circumference was measured through a point in the umbilicus using non-stretchable measuring tape; measurements were taken in the horizontal plane at the end of a normal expiration, and recorded to the nearest 0.1 cm, with the patient standing feet shoulder-length apart and with a relaxed abdomen. When the participants were sitting, systolic and diastolic blood pressure were measured after they had rested for at least 5 min. Two readings were acquired with 15-minute intervals, using a calibrated mercury sphygmomanometer appropriate for adult cuff size, and the final average blood pressure was taken for analysis.

### Laboratory Assessments and Biochemical Measurements

Participants underwent an overnight fasting period lasting at least 8 h before having venous blood samples collected. The hospital laboratory processed samples upon collection and placed them in sterile containers. Serum total cholesterol, triglycerides, low-density lipoprotein (LDL) cholesterol, and high-density lipoprotein (HDL) cholesterol levels were measured via standardized enzymatic colorimetric methods. Fasting glucose was measured via the glucose oxidase method. Insulin levels and HOMA-IR were not assessed due to resource constraints and protocol focus on IDF-defined MetS components. All laboratory assays, including method and quality control protocols, were performed by trained professionals in compliance with hospital laboratory standards.

### IBS Diagnosis and Classification

The diagnosis of IBS was made if the patient had recurrent abdominal pain that occurred on average at least once per week in the past three months, with a minimum of 6 months from the time of diagnosis. In addition, the symptoms met at least two of these criteria in the past three months: change in frequency of defecation, altered consistency of stool, and symptoms with regard to defecation. Patients were categorized into subgroups based on the Bristol Stool Chart: these four subgroups include mixed IBS (IBS-M), constipation-predominant IBS (IBS-C), diarrhea-predominant IBS (IBS-D), and unspecified IBS (IBS-U).

### Metabolic Syndrome Definition

MetS was defined according to the International Diabetes Federation 2005/2006 consensus criteria [15]. In addition to having abdominal obesity (waist circumference ≥ 94 cm for men and > 80 cm for women), patients were classified as having MetS if they satisfied two or more of the following four criteria: (1) TG ≥ 150 mg/dL or specific treatment for dyslipidemia, (2) the presence of type 2 diabetes mellitus or a fasting blood glucose level ≥ 100 mg/dL, (3) hypertension (≥ 130 mm Hg systolic or ≥ 85 mm Hg diastolic or on hypertension prescription), and (4) a low HDL cholesterol level (< 50 mg/dL females in and < 40 mg/dL in males). The International Diabetes Federation (IDF) consensus recommends the use of ethnic‑ or country‑specific thresholds whenever available, and, for Eastern Mediterranean and Middle Eastern populations, to use the Europid cutoffs.

### Statistical Analysis

Data were analyzed using standard statistical methods. All analyses were performed using SPSS Version 26. Continuous variables were first assessed for normality and are presented as mean ± standard deviation or median (interquartile range), as appropriate. Categorical variables are summarized as frequencies and percentages. Continuous variables were compared using the independent t-test or the Mann–Whitney U test, depending on data distribution, while categorical variables were compared using the chi-square test or Fisher’s exact test.

### Assessment of Association

Univariate and multivariate binary logistic regression tests were conducted to examine the link between IBS and metabolic syndrome. The selection process for covariates in the multivariate logistic regression models followed established biological plausibility criteria and risk factors of IBS and MetS from current literature. Accordingly, three sequential models were developed to adjust for possible confounding variables:

- Model 1: Unadjusted (crude) association.
- Model 2: Adjusted for demographic factors (age and sex).
- Model 3: Fully adjusted for demographics and lifestyle/anthropometric factors (age, sex, BMI, smoking status, marital status, and residence); waist circumference, a MetS component, was excluded from modeling as the outcome was the composite IDF-defined MetS diagnosis. Marital status was included as a social determinant potentially associated with diet, physical activity, stress, and healthcare use, all of which can influence MetS risk [16, 17]. To explore factors independently associated with metabolic syndrome within the IBS cohort, initial univariate analyses were conducted to compare demographic, lifestyle, and clinical characteristics between IBS patients with and without metabolic syndrome. Multivariable binary logistic regression analysis was performed using a forced-entry approach. Results are presented as adjusted odds ratios (AORs) with 95% confidence intervals. A two-sided p-value < 0.05 was considered statistically significant. The study reports results as Odds Ratios (OR) with 95% Confidence Intervals (CI). The final model’s goodness-of-fit was determined by the Hosmer-Lemeshow test, which shows adequate fit when the p-value > 0.05. The models’ discrimination capacity was measured through the Area Under the Receiver Operating Characteristic (ROC) curve (AUC). An AUC value of 0.7 to 0.8 represents acceptable discrimination, while an AUC above 0.8 indicates excellent results. Variance inflation factors (VIF 1.055–3.298) confirmed the absence of multicollinearity.

## Results

Table 1 presents baseline characteristics of 140 non-IBS and 140 IBS participants, showing significant differences in cardiometabolic risk factors and demographics. Based on self-reported data, none of the participants reported adherence to any structured dietary pattern. The research identified that IBS patients exhibited higher rates of diabetes and hypertension medications intake, IBS family history, comorbidities, and metabolic syndrome features, including high FBG, BP, TG, and WC. IBS patients were numerically older than controls (median 31.5 vs. 27 years; *p* = 0.418), an unexpected direction that may conservatively bias observed MetS associations given age as a cardiometabolic risk factor. No significant sex difference was observed (*p* = 0.145). The groups showed no significant differences in residence, occupation, marital status, and education, and BMI and HDL levels. Non-IBS individuals showed a higher smoking rate when compared to IBS patients (11.4% vs. 3.6%, *p* = 0.013). Family history of IBS was far more common in cases (41.4% vs. 7.9%, *p* < 0.001). Metabolic syndrome prevalence was higher in IBS patients (49.3% vs. 17.1%, *p* < 0.001), driven by a higher proportion of central obesity (71.4% vs. 44.3%), FBG ≥ 100 mg/dL (42.9% vs. 19.3%), and BP ≥ 130/85 (57.9% vs. 17.9%; all *p* < 0.001). Waist-to-height ratio was significantly higher in IBS patients compared to the control. Despite exclusion of late-onset cases (≥ 50 years), IBS patients showed substantially accelerated cardiometabolic risk with elevated MetS components, including hypertension (57.9% vs. 17.9%) and dysglycemia (42.9% vs. 19.3%). The IBS groups showed elevated LDL-C levels at 115 mg/dL versus 102 mg/dL (*p* = 0.01) and with borderline total cholesterol differences. No disparities appeared in HDL-C or triglycerides medians, though TG ≥ 150 was significantly higher in IBS patients (25.7% vs. 15.7%, *p* = 0.039).

**Table 1:** Demographic, clinical, and laboratory characteristics of the study cohorts

Parameter	non-IBS (*N*, % / median, IQR)	IBS cases (*N*, % / median, IQR)	Sig.
Number of participants	140	140	
Age (years)	27 (23–41)	31.5 (23–46)	0.418
Sex Females Males	76 (54.3%) 64 (45.7%)	88 (62.9%) 52 (37.1%)	0.145
Residence Rural Urban	22 (15.7%) 118 (84.3%)	33 (23.6%) 107 (76.4%)	0.098
Smoking status Non-smoker Current smoker	124 (88.6%) 16 (11.4%)	135 (96.4%) 5 (3.6%)	0.013*
Occupation Unemployed employed	30 (21.4%) 110 (78.5%)	41 (29.3%) 99 (70.7%)	0.3
Marital state Single Married	76 (54.3%) 64 (45.7%)	72 (51.4%) 68 (48.6%)	0.901
Drug history Diabetes mellitus Rx Hypertension Rx Dyslipidemia Rx	8 (4.3%) 4 (2.1%) 0 (0%)	22 (23.7%) 18 (19.4%) 2 (2.2%)	< 0.001* < 0.001* 0.04*
Education level Illiterate Educated	17 (12.1%) 123 (87.8%)	23 (16.4%) 117 (83.6%)	0.424
Self-reported family history of IBS	11 (7.9%)	58 (41.4%)	< 0.001*
Comorbidities Hypertension Diabetes	9 (6.4%) 13 (9.3%)	20 (14.3%) 19 (13.6%)	0.031* 0.260
Metabolic syndrome (MetS)	24 (17.1%)	69 (49.3%)	< 0.001*
FBG ≥ 100 mg/dl	27 (19.3%)	60 (42.9%)	< 0 0.001 *
BP ≥ 130/85	25 (17.9%)	81 (57.9%)	< 0 0.001 *
MetS. HDL (< 50 mg/dL in females, and < 40 mg/dL in males)	53 (37.9%)	53 (37.9%)	1.000
MetS. TG ≥ 150 mg/dL	22 (15.7%)	36 (25.7%)	0.039*
MetS. WC(≥ 94 cm in males and > 80 cm in females)	62 (44.3%)	100 (71.4%)	< 0.001*
Waist circumference (cm)	95 (81-106.75)	103 (90.25–116)	< 0.001*
Waist-to-height ratio	0.56 (0.49–0.56)	0.62 (0.54–0.71)	< 0.001*
BMI (kg/m^2)	28.3 (24.7–33.5)	27.8 (24.1–32.9)	0.879*
Mean arterial pressure (mmHg)	93.3 (86.7–93.3)	96.7 (93.3–106. 7)	< 0.001*
Serum total Cholesterol (mg/dl)	175 (146-208.25)	186.5 (156-215.5)	0.06
Serum LDL-C (mg/dl)	102 (76–132)	115 (91-139.5)	0.01*
Serum HDL-C (mg/dl)	48 (40-56.75)	48 (41–53)	0.539
Serum Triglycerides (mg/dl)	100 (68.25-133.25)	110.5 (68.5–150)	0.353
Fasting Blood glucose (mg/dl)	90 (80-96.75)	93.5 (85–102)	0.003*

Notes: Sig. = significance (p-value). BMI is body mass index, FBG (fasting blood glucose component), BP (blood pressure component), TG (triglyceride component), HDL (high-density lipoprotein component), and LDL (low-density lipoprotein). * Significant p-value. IBS-C: irritable bowel syndrome constipation type; IBS-D: irritable bowel syndrome diarrhea type; IBS-M: irritable bowel syndrome mixed type; IBS-U: irritable bowel syndrome unspecified type. DM: diabetes mellitus. None of the participants reported adherence to any structured dietary pattern

Table 2 shows the comparative characteristics between IBS patients with and without metabolic syndrome. Patients with metabolic syndrome were significantly older, with a median age of 42 years (IQR: 26–50) compared to 23 years (IQR: 22–37 years) in those without metabolic syndrome (*p* < 0.001). Sex distribution did not differ significantly between groups (*p* = 0.631), nor did residence (*p* = 0.489), or smoking status (*p* = 0.429). Concerning marital status, 65.2% of IBS patients with metabolic syndrome were married versus only 32.4% in those without metabolic syndrome (< 0.001). Medication history differed significantly, with 21.7% vs. 2.8% diabetes treatment and 17.4% vs. 2.8% hypertension treatment in MetS vs. non-MetS IBS patients. Among IBS patients with MetS, 76.8% had formal education compared to 90.1% without MetS (*p* < 0.001). Compared to those without MetS, IBS patients with MetS showed higher BMI, with a median of 31.25 kg/m^2^ (IQR: 27.8–35.4, *p* < 0.001), a median serum total cholesterol of 199 mg/dl (174.5-222 mg/dl, *p* < 0.001), a median serum LDL-C of 120 mg/dl (102–149 mg/dl, *p* = 0.01).

**Table 2:** Demographic, clinical, and laboratory characteristics of those without metabolic syndrome and those with metabolic syndrome in the study cohort

Parameter	Non-Metabolic syndrome (*N*,% / median, IQR)	Metabolic syndrome (NN,% / median, IQR)	Sig.
Number of participants	71	69	
Age (years)	23 (22–37)	42 (26–50)	< 0.001*
Sex Females Males	46 (64.8%) 25 (35.2%)	42 (60.9%) 27 (39.1%)	0.631
Residence Rural Urban	15 (21.1%) 56 (78.9%)	18 (26.1%) 51 (73.9%)	0.489
Smoking Status Non smoker Current smoker	70 (98.6%) 1 (1.4%)	65 (94.2%) 4 (5.8%)	0.429
Occupation Unemployed Employed	16 (22.5%) 55 (77.5%)	25 (36.2%) 44 (63.8%)	0.082
Marital state Single Married	48 (67.6%) 23 (32.4%)	24 (34.8%) 45 (65.2%)	< 0 0.001*
Drug history DM Rx Hypertension Rx Dyslipidemia Rx ^FF^ Antispasmodic Rx	2 (2.8%) 2 (2.8%) 0 (0%) 15 (21.1%)	15 (21.7%) 12 (17.4%) 2 (2.9%) 15 (21.7%)	< 0.001* 0.004* 0.241 0.93
Education level Illiterate Educated	7 (9.9%) 64 (90.1%)	16 (23.2%) 53 (76.8%)	< 0.001*
Self-reported family history of IBS	32 (45.1%)	26 (37.7%)	0.375
IBS type IBS-C IBS-D IBS-M IBS-U	48 (67.6%) 10 (14.1%) 9 (12.7%) 4 (5.6%)	43 (62.3%) 10 (14.1%) 8 (11.6%) 8 (11.6%)	0.651
BMI (kg/m^2)	25.35 (23.2–28.6)	31.25 (27.8–35.4)	< 0.001*
Mean arterial pressure (mmHg)	93.3 (86.7–100)	103.3 (96.7–110)	< 0.001*
Serum total Cholesterol (mg/dl)	170 (142–200)	199 (174.5–222)	< 0.001*
Serum LDL-C (mg/dl)	109 (82–125)	120 (102–149)	0.01*

Notes:  Sig. = significance (p-value). * Significant p-value

Table 3 presents a comparative analysis of metabolic syndrome and its components among IBS subtypes. The prevalence of abdominal obesity increased across the subtypes, ranging from 65.9% in IBS-C to 91.7% in IBS-U; however, this finding did not reach statistical significance (*p* = 0.181). The prevalence of diabetes mellitus between subtypes was non-significant, with 58.3% in IBS-U individuals. Low HDL cholesterol was present in 33% of patients with IBS-C and in 60% of patients with IBS-D (*p* = 0.157), while high triglycerides were found in 15% IBS-D patients and 41.7% IBS-U patients (*p* = 0.154). The prevalence of hypertension was maximum in IBS-U at 83.3% and uniform across subtypes with (54.9–83.3%, *p* = 0.31). There were no significant differences (*p* = 0.651) in the overall prevalence of metabolic syndrome, which was 47.3% (IBS-C), 50% (IBS-D), 47.1% (IBS-M), and 66.7% (IBS-U).

**Table 3:** Distribution of metabolic syndrome and its components according to IBS subtypes

Parameters	IBS-C (*N*, %)	IBS-D (*N*, %)	IBS-M (*N*, %)	IBS-U (*N*, %)	*p*-value
Abdominal obesity	60/91 (65.9%)	15/20 (75%)	14/17 (82.4%)	11/12 (91.7%)	0.181
Diabetes mellitus	39/91 (42.9%)	8/20 (40%)	6/17 (35.3%)	7/12 (58.3%)	0.651
*High Triglycerides	21/91 (23.1%)	3/20 (15%)	7/17 (41.2%)	5/12 (41.7%)	0.154
*Low HDL- cholesterol	30/91 (33%)	12/20 (60%)	6/17 (35.3%)	5/12 (41.7%)	0.157
Hypertension	50/91 (54.9%)	11/20 (55%)	10/17 (58.8%)	10/12 (83.3%)	0.31
Metabolic syndrome	43/91 (47.3%)	10/20 (50%)	8/17 (47.1%)	8/12 (66.7%)	0.651

*Low or high cut-offs are defined by the specific diagnostic thresholds of international diabetic federation criteria (IDF)

Table 4 presents the results of the univariate and multivariate logistic regression analyses evaluating the association between IBS and MetS. In the unadjusted model (Model 1), IBS was significantly associated with increased odds of metabolic syndrome (OR: 4.697; 95% CI: 2.709–8.145; *p* < 0.001). The association between the variables stayed statistically significant and robust when demographic variables were adjusted in Model 2 (age and sex), which showed an increased odds ratio of 5.932 (95% CI: 3.175–11.085; *p* < 0.001). Model 3, which included adjustments for age and sex, BMI, smoking status, and residence, showed that IBS status remained a strong independent predictor of metabolic syndrome. The odds of developing metabolic syndrome were eight times higher in IBS patients than in the control group (OR: 7.818; 95% CI: 3.913–15.620; *p* < 0.001). Regarding covariates in the final model, older age (OR: 1.053; *p* = 0.002) and higher BMI (OR: 1.155; p 0.001) were significantly associated with higher odds of metabolic syndrome. The odds of developing MetS were lower for females than males (OR: 0.395; 95% CI: 0.195–0.803; *p* = 0.010). There was no statistical significance between smoking status and rural or urban residence (*p* > 0.05). The model achieved better discriminatory performance through the inclusion of additional variables. The Area Under the Curve (AUC) increased from 0.681 (95% CI: 0.615–0.747) in the crude model to 0.852 (95% CI: 0.806–0.898) in the fully adjusted model, indicating excellent discrimination. The Hosmer-Lemeshow test for the final model yielded a p-value of 0.442, suggesting good calibration and adequate model fit.

**Table 4:** Logistic regression analysis for the association between IBS and metabolic syndrome

Variable	Model 1		Model 2		Model 3	
	OR (95% CI)	*p*-value	OR (95% CI)	*p*-value	OR (95% CI)	*p*-value
IBS status (ref: controls)	4.697 (2.709–8.145)	< 0.001*	5.932 (3.175–11.085)	< 0.001*	7.818 (3.913–15.620)	< 0.001*
Age (years)	-	-	1.095 (1.065–1.126)	< 0.001*	1.053 (1.020–1.088)	0.002*
Sex (ref: males)	-	-	0.472 (0.250–0.891)	0.021*	0.395 (0.195–0.803)	0.010*
BMI (kg/m^2^)	-	-	-	-	1.155 (1.084–1.231)	< 0.001*
Smoking (ref: never smoking)	-	-	-	-	1.009 (0.276–3.693)	0.989
Residence (ref: rural)	-	-	-	-	0.667 (0.316–1.406)	0.287
Model fit (Hosmer-Lemeshow)						0.442
AUC (95% CI)	0.681 (0.615–0.747)	< 0.001*	0.821 (0.77–0.872)	< 0.001*	0.852 (0.806–0.898)	< 0.001*

OR: Odds Ratio; CI: confidence interval; * statistically significant, AUC: area under the curve

Model 1: crude association

Model 2: adjusted for age and sex

Model 3: adjusted for age, sex, BMI, smoking, and residence

Binary logistic regression analysis was run to ascertain the effects of age, sex, smoking status, BMI, family history of IBS, and marital status on the likelihood of metabolic syndrome. In the univariate analysis, only age, BMI, and marital status were statistically significant, as shown in Table 5. The multivariate regression model revealed that only BMI was a statistically significant independent predictor of metabolic syndrome development. The model correctly classified 75.7% of the cases with 73.9% sensitivity and 77.5% specificity, with a positive predictive value (PPV) of 76.1% and a negative predictive value (NPV) of 75.3%. Among the six predictor variables, only one was a statistically significant independent predictor: a one-unit increase in BMI had 18% greater odds of having metabolic syndrome (AOR 1.18, 95% CI: 1.08–1.3, *p* < 0.001) (Table 5). The Hosmer-Lemeshow goodness-of-fit test confirmed adequate model calibration (*p* = 0.134). The final multivariate logistic regression model was highly significant overall (omnibus test: χ² = 43.944, df = 6, *p* < 0.001) and accounted for 35.9% of the variance in the presence of metabolic syndrome among IBS patients (Nagelkerke R² = 0.359). The model showed good discriminative performance, with an area under the receiver operating characteristic (ROC) curve of 0.813, suggesting that it effectively distinguishes between IBS patients with and without metabolic syndrome.

**Table 5:** Univariate and multiple logistic regression analyses of independent predictors of metabolic syndrome in IBS patients

Predictor	Univariable				Multivariable				
	Sig.	COR	95% CI		Sig	AOR	95% CI		VIF
			Lower	Upper			Lower	Upper	
Age (years)	< 0.001*	1.074	1.041	1.108	0.311	1.030	0.973	1.090	3.298
Sex (ref: females)	0.631	1.183	0.596	2.349	0.069	2.198	0.939	5.143	1.1
Smoking status (ref: non-smoking)	0.219	1.348	0.837	2.169	0.106	7.514	0.650	86.820	1.065
BMI (kg/m^2)	< 0.001*	1.2	1.114	1.292	< 0.001*	1.18	1.082	1.300	1.605
Self-reported family history of IBS (ref: negative)	0.375	0.737	0.375	1.447	0.802	1.110	0.490	2.514	1.055
Marital status (ref: single)	< 0.001*	3.913	1.940	7.894	0.806	1.173	0.329	4.184	2.795
Model fit (Hosmer-Lemeshow)					0.134				

COR: crude odds ratio; AOR: adjusted odds ratio; CI: confidence interval; * significant p-value

Table 6 presents the distribution and odds of metabolic syndrome by body mass index categories in IBS patients. Metabolic syndrome was present in 6of 38 participants with normal BMI (25 kg/m^2^), 18 of 40 who were overweight (25–29.9 kg/m^2^), and 45 of 62 who were obese (≥ 30 kg/m^2^). Compared with normal-weight participants, overweight IBS individuals had about a fourfold higher odds of metabolic syndrome (4.364, 95% CI: 1.494–12.742, *p* = 0.007). Obese IBS participants had approximately a fourteenfold higher odds of metabolic syndrome than the normal-weight group (14.118, 95% CI: 5.014–39.75, *p* < 0.001).

**Table 6:** Association between metabolic syndrome and BMI categories in this IBS cohort

BMI categories	Total *N* (%)	Metabolic syndrome present *N* (%)	OR	95% CI	*p*-value
Number of participants	140	69			
Normal weight (< 25 kg/m^2^)	38 (27.1%)	6 (8.7%)	Reference	-	-
Overweight (25–29.9 kg/m^2^)	40 (28.6%)	18 (26.1%)	4.364	1.494–12.742	0.007*
Obese (≥ 30 kg/m^2^)	62 (44.3%)	45 (65.2%)	14.118	5.014–39.75	< 0.001*

*Statistically significant

## Discussion

This case-control study showed a robust independent association between irritable bowel syndrome and metabolic syndrome because IBS patients showed almost three times higher metabolic syndrome prevalence. Higher-than-expected MetS prevalence vs. Turkish reference reflects Egypt’s accelerating cardiometabolic epidemic, tertiary referral patterns, and stringent Rome IV case ascertainment [10]. The multivariate logistic regression analysis showed IBS as an independent predictor of metabolic syndrome after adjusting for demographic and lifestyle factors. The predictive model showed strong discrimination and adequate calibration, which demonstrates the substantial cardiometabolic impact of this functional gastrointestinal disorder. The only independent predictor of metabolic syndrome co-occurrence among IBS patients was elevated BMI levels. Notably, none of the participants reported adherence to structured dietary patterns, which may represent a missed opportunity for targeted lifestyle interventions in this high-risk group.

An important descriptive finding of this study is the atypical IBS subtype distribution, with nearly half of patients classified as IBS-C, and lower proportions of IBS-D and IBS-M, whereas most population-based studies report IBS-D and IBS-M as the most common subtypes [18]. This pattern likely reflects a combination of tertiary-care referral dynamics, regional dietary factors (high refined-carbohydrate, low-fiber patterns in the Nile Delta), and potential diagnostic or recording bias favoring constipation-predominant symptomatology in routine clinical practice.

Our findings align with and extend the conclusions of a recent 2025 systematic review and meta-analysis synthesizing data from 49,662 individuals across 10 observational studies [10]. This reflects population-specific characteristics, along with sampling methods and the epidemiological situation of Mediterranean and Middle Eastern regions in our cohort. The elevated risk level holds clinical significance because previous cross-sectional studies conducted among East Asian populations, including Korea, Japan, and China, demonstrated that IBS patients had 2.01 to 4.70 times higher metabolic syndrome rates than control groups [3, 10]. The consistency across geographically diverse populations supports the biological plausibility of IBS as a cardiometabolic risk factor.

Our research demonstrated a particular set of individual metabolic irregularities. A substantially higher abdominal obesity rate in IBS patients was supported by the meta-analytic findings about growing abdominal obesity risk and expanded waist circumference in IBS [10]. A study that used computed tomography (CT) imaging to measure visceral adipose tissue (VAT) and subcutaneous adipose tissue (SAT) directly to establish more accurate quantification than anthropometric methods alone [19]. The multivariate analysis demonstrated that visceral adiposity measured by VAT area, VAT/SAT ratio, and waist circumference was independently associated with increased IBS risk [19]. Yu S et al. conducted a study which showed that subjects in the highest quartile experienced an 8–35% high risk of IBS development compared to the reference group [20].

Notably, overall BMI did not differ significantly between groups, suggesting a compartmentalized distribution of adiposity in which IBS patients develop preferential visceral fat accumulation despite comparable total body mass—a phenomenon previously documented by Lee et al., who demonstrated that visceral adiposity, but not subcutaneous fat, was associated with increased IBS risk [19]. Although overall BMI was comparable between groups, IBS cases exhibited disproportionate central obesity, which created collider bias in the crude estimates. The findings from our study gain additional significance because BMI was independently associated with a higher risk of metabolic syndrome among IBS patients, indicating that weight may act as a secondary metabolic stressor in this population. The current study demonstrates a dose-response relationship within the IBS cohort, with overweight individuals showing fourfold higher odds and obese individuals showing fourteenfold higher odds of metabolic syndrome compared to normal-weight IBS participants. The exponential escalation shows that IBS patients experience an inherent level of cardiometabolic risk, which results from visceral adiposity and inflammation caused by gut dysbiosis. Yet BMI-driven weight gain adds a multiplicative effect to this risk. The strong and graded association between BMI categories and metabolic syndrome in this IBS cohort highlights obesity as a central variable associated with metabolic risk in IBS rather than IBS itself being inherently metabolic [21]. IBS remained independently associated with a higher prevalence of cardiometabolic abnormalities after adjustment for obesity and other covariates. The odds ratio amplification after BMI adjustment indicates positive confounding. Full adjustment isolates IBS-specific cardiometabolic risk, consistent with meta-analytic findings despite population difference [10]. Notably, the odds ratio for the association between IBS and MetS increased from 4.697 in the crude model to 5.932 after adjustment for age and sex. This pattern reflects negative confounding by demographics: IBS patients in this cohort were relatively young, and younger age is associated with a lower baseline risk of MetS. Consequently, the unadjusted model underestimates the strength of the IBS–MetS association, and adjusting for age removes this protective age effect, revealing a stronger underlying relationship. The finding that only BMI independently predicted metabolic syndrome in the IBS group suggests that glycemic deterioration in this cohort is adiposity-mediated.

Glycemic parameters revealed elevated fasting blood glucose and elevated prevalence of impaired fasting glucose ≥ 100 mg/dL in IBS patients, although the absolute diabetes prevalence remained similar between groups. This selective elevation of fasting blood glucose without frank diabetes is instructive. The combination of these factors shows that IBS patients may develop insulin resistance and dysglycemia before they progress to type 2 diabetes mellitus, which matches the metabolic characteristics of pre-diabetes. IBS was associated with insulin resistance–compatible features (higher fasting glucose and impaired fasting glycemia), suggesting a possible link with dysglycemia that requires confirmation in longitudinal studies. Our study found that diabetes medication use was significantly elevated in IBS patients, yet diabetes prevalence by diagnostic criteria remained similar. The conflicting results may suggest the pre-diabetic metabolic condition of IBS patients because they develop insulin resistance before overt hyperglycemia or diabetes mellitus, which differs from the diabetes-related patterns that may occur in people with severe obesity. The meta-analysis revealed no significant association between IBS and the prevalence of diabetes or fasting blood glucose levels [10]. A potential point of disagreement with select literature concerns the marginal or absent associations of IBS with frank diabetes in some studies.

The results of our study showed abnormal lipid profiles, which included higher median LDL cholesterol and increased prevalence of triglycerides ≥ 150 mg/dL and borderline raised total cholesterol levels. These results match the meta-analysis findings, which showed IBS patients have elevated total cholesterol, LDL, and triglycerides along with decreased HDL cholesterol [10]. Our study found no difference in HDL levels between groups, which contrasts with this meta-analytic summary. The younger age of our participants (IBS: 31.5 years), along with the greater number of smokers in the control group, explains this result because smoking influences HDL metabolism. This finding in our cohort matches the findings from Moosazadeh et al. and Momayyezi et al., who discovered through cross-sectional research that smoking is associated with lower HDL levels [22, 23]. Blood pressure abnormalities were prominent: the prevalence of elevated blood pressure (≥ 130/85 mmHg) in IBS patients dwarfed the control prevalence, with mean arterial pressure 3.4 mmHg higher in IBS cases. Our findings substantiate the meta-analytic evidence that IBS patients develop systolic hypertension [10].

IBS in this cohort appeared to cluster with a distinct metabolic profile characterized by abdominal obesity, dyslipidemia, and dysglycemia that needs dual treatment strategies that involve metabolic assessment and risk evaluation for all patients, plus intensive weight management for IBS patients who are overweight or obese, since their weight loss results surpass those of people without IBS. However, Yau et al. explicitly concluded: “excess body weight may not be a primary driver of IBS risk” [24]. This review examined 21 cross-sectional studies, 5 case-control studies, and 1 cohort study conducted in Europe, Asia, North America, and the Middle East [24]. The meta-analysis revealed one essential exception because studies that applied Rome IV diagnostic criteria found a significant link between obesity and IBS; however, Rome III studies did not detect any connection.

Conversely, research by Javadekar et al. and Dogan et al. found no statistically significant associations between IBS and metabolic syndrome, or its components, including abdominal obesity [25, 26]. The different results likely stem from differences in diagnostic standards, IBS type distribution, metabolic syndrome criteria, and population-specific factors. Marital status differed substantially between IBS patients with and without MetS, likely reflecting age and socioeconomic patterning. Married status correlates with older age and potentially greater family-related cardiometabolic risk factors (dietary patterns, sedentary behavior, and chronic stress) [27]. Although not included in multivariable models due to collinearity with age and event numbers, this deserves consideration as a socioeconomic modifier in future studies. The observed MetS excess that persists after age/SES adjustment suggests that the IBS-MetS association transcends these traditional risk pathways.

While our study demonstrates significant associations between IBS and adverse cardiometabolic profiles, the underlying biological mechanisms remain hypothesized based on prior literature. For example, studies have proposed that IBS-related gut dysbiosis, visceral adiposity, corticotropin-releasing factor pathways, and altered gut permeability may contribute to cardiometabolic dysregulation [28–30]. However, these pathways were not directly assessed in our cohort, and their precise role in linking IBS and MetS requires confirmation through mechanistic and longitudinal studies. Clinical evidence suggests that metabolic syndrome may occur at relatively high rates even among younger IBS patients, which makes it necessary to perform regular cardiometabolic assessments that include BMI evaluation, waist measurement, and blood pressure, fasting glucose, and lipid profile tests at IBS clinics for overweight and obese patients [10, 31].

The detection of metabolic syndrome in IBS patients at an early stage creates a chance to apply lifestyle changes, which involve diet adjustments, physical exercise, and weight management, to enhance both digestive health and life quality and heart disease prevention [10, 30]. A series of longitudinal studies should be conducted to monitor metabolic factors and microbiota composition, thereby establishing causal relationships between these variables. Multisite studies with longer follow-up across diverse Egyptian and regional populations are needed to validate these findings. Population-based studies would complement these findings from specialist settings. Future studies should employ 1:1 matching on age, sex, and BMI to complement regression-based approaches and strengthen causal inference. Future studies using 16 S rRNA gene sequencing or shotgun metagenomics can be designed to strengthen causal inference. Late-onset IBS-MetS patterns remain unexamined and warrant future investigation. Elevated FBG and the prevalence of diabetes medications provide indirect evidence, but future studies should incorporate fasting insulin, HbA1c, and OGTT to enable comprehensive glycemic phenotyping. Psychiatric comorbidity may amplify cardiometabolic risk through shared stress-inflammation pathways; future studies should include psychiatrically stratified analyses to examine effect modification. The potential residual confounding by SES should be acknowledged, and future studies with larger sample sizes should incorporate more granular socioeconomic measures into multivariable models.

### Clinical and Policy Implications

The study has significant implications for both clinical practice and policy. With such a large increase in odds of having metabolic syndrome in individuals with IBS, consideration should be made for routine cardiometabolic screening in IBS patients, especially those who are either overweight or obese. In order to establish this routine screening for IBS patients in clinical settings, clinicians may need to include the assessment of metabolic status (e.g., blood pressure, fasting glucose, lipid profile, or waist circumference) in the standard protocol for management of IBS, rather than only focusing on symptoms primarily involving the gastrointestinal tract.

From a health systems perspective, these data support a more integrated and coordinated multidisciplinary model of care that includes collaboration between gastroenterology and metabolic medicine. The early detection of high-risk patients with IBS would provide opportunities to initiate timely interventions for lifestyle modification interventions including weight management, dietary changes, and increased physical activity to potentially reduce the long-term burden of cardiovascular disease. At a policy level, inclusion of IBS into risk stratification of metabolic syndrome may be especially relevant in regions where there is a high prevalence of both conditions. Public health awareness campaigns should emphasize the extra-intestinal risks associated with IBS and encourage preventive screening programs to be incorporated into primary healthcare delivery systems. Future revisions to clinical practice guidelines may advocate for cardiometabolic assessments to be included in routine management of IBS.

### Limitations

The research has multiple restrictions that need to be acknowledged. The cross-sectional ascertainment of exposures and outcomes within this case-control framework prevents the identification of causality and temporal sequence; researchers cannot determine whether IBS leads to the development of metabolic syndrome, whether environmental factors are responsible for both conditions, or whether early metabolic problems result in IBS symptoms by disrupting protective barriers. The study did not directly measure inflammatory biomarkers (CRP, IL-6, TNF-α, LPS, HOMA-IR as a proxy for insulin resistance) in its cohort, so investigators had to rely on existing literature rather than examine actual inflammatory dysregulation in their patients. This study was conducted at a single tertiary care center (Mansoura Specialized Medical Hospital, Dakahlia, Egypt) over a 6-month period, which may limit the generalizability of findings to other geographic regions, healthcare settings, or populations with different epidemiological profiles. The relatively short enrollment window may not fully capture seasonal variations in patient presentations or metabolic parameters. Moreover, Consecutive non-probability sampling from GI outpatient clinics may overrepresent moderate-to-severe IBS cases typical of specialist referral patterns. As a tertiary care center, the study may underrepresent mild community-managed IBS cases, potentially inflating specialist-confirmed prevalence. However, comparable baseline cardiometabolic profiles between GI and internal medicine clinic patients minimize referral bias for metabolic syndrome assessment. Population-based studies would complement these findings from specialist settings. The unmatched design, while statistically controlled through regression adjustment, lacks the individual matching that could further minimize residual confounding. The study did not systematically collect detailed dietary and physical activity data, which prevents researchers from evaluating how lifestyle factors can be modified to affect both IBS and metabolic parameters. Gastrointestinal microbiome composition was not evaluated, although dysbiosis is hypothesized to be a core mechanism. Excluding late-onset IBS (≥ 50 years) created a younger cohort (median age 31.5 years) that excludes age-typical MetS progression. The observed high prevalence of MetS (49.3%) in this younger population strengthens the association between IBS and MetS, suggesting accelerated cardiometabolic risk. The IBS subtype distribution was skewed toward IBS‑C, with very small numbers in the IBS‑U group, rendering subtype‑specific analyses underpowered; percentages for MetS and its components within subtypes, especially IBS‑U, should therefore be interpreted cautiously as descriptive rather than inferential. Pre-enrollment, subjects with an organic history of gastrointestinal disease, inflammatory bowel disease, celiac disease, or diabetes who require insulin have been excluded; this may have led to enrichment for simpler phenotypes of IBS and metabolic syndrome and to an underestimate of disease severity and comorbidity in the larger population. Moreover, the lack of measurements of adipokines (leptin, adiponectin, resistin) and markers of barrier dysfunction (zonula occludens-1, fecal calprotectin, serum intestinal fatty acid-binding protein) made direct assessment of the proposed mechanisms impossible. Insulin levels and HOMA-IR were not measured, limiting direct quantification of insulin resistance. Exclusion of patients with any psychiatric disorders (prevalence 30–50% in IBS) removes a phenotypically relevant subgroup with established IBS-anxiety/depression comorbidity. This enhances diagnostic purity but limits generalizability to unselected clinical IBS populations. Waist circumference cutoffs followed IDF Europid thresholds (≥ 94 cm for men, ≥ 80 cm for women), as currently recommended for Middle Eastern populations, pending validated ethnicity‑specific values. Although education level differed between IBS patients with and without MetS, suggesting a possible socioeconomic gradient, we did not include education (or broader SES indicators) in the multivariable models. Our adjustment set focused on established clinical and lifestyle confounders (age, sex, BMI, smoking, residence), and adding additional, correlated variables risked model overfitting given the number of events. Obese IBS patients had substantially higher MetS odds compared to normal-BMI IBS patients. However, the very wide CI reflects limited precision due to only 6 MetS cases among 38 normal-BMI IBS patients, characteristic of sparse data in the reference category.

### Concluding Perspective

This study provides robust epidemiological evidence that IBS represents a significant and independent risk factor for metabolic syndrome, with an adjusted odds ratio of 7.82. The association is accompanied by cardiometabolic abnormalities spanning dyslipidemia, dysglycemia, hypertension, and preferential abdominal adiposity. The analysis of IBS patients shows that BMI serves as the main factor predicting metabolic syndrome development. Yet, IBS status maintains its independent influence on metabolic syndrome risk according to multiple variable models, which indicates that additional disease-specific mechanisms affect this population apart from obesity. These findings support systematic cardiometabolic screening in IBS populations, evaluation of dual-benefit lifestyle and pharmacologic interventions targeting both gastrointestinal and metabolic homeostasis, and longitudinal studies to clarify causality and identify optimal prevention strategies. Ultimately, recognition of IBS as a cardiometabolic risk state may reshape clinical management paradigms, integrating gastroenterologic and internistic perspectives to improve long-term health outcomes in this highly prevalent population.

## Data Availability

The relevant authors of the current study have kindly provided the datasets under analysis upon appropriate request from the corresponding author.
